# Atmospherically deposited elemental mercury drives evasion of mercury from the ocean and freshwaters

**DOI:** 10.1093/nsr/nwaf590

**Published:** 2025-12-26

**Authors:** Xuewu Fu, Hui Zhang, Kaihui Tang, Jonas Sommar, Jen-How Huang, Zhengcheng Song, Yanxu Zhang, Charles T Driscoll, Xinbin Feng

**Affiliations:** State Key Laboratory of Environmental Geochemistry, Institute of Geochemistry, Chinese Academy of Sciences, Guiyang 550081, China; State Key Laboratory of Environmental Geochemistry, Institute of Geochemistry, Chinese Academy of Sciences, Guiyang 550081, China; State Key Laboratory of Environmental Geochemistry, Institute of Geochemistry, Chinese Academy of Sciences, Guiyang 550081, China; University of Chinese Academy of Sciences, Beijing 100049, China; State Key Laboratory of Environmental Geochemistry, Institute of Geochemistry, Chinese Academy of Sciences, Guiyang 550081, China; State Key Laboratory of Environmental Geochemistry, Institute of Geochemistry, Chinese Academy of Sciences, Guiyang 550081, China; School of Atmospheric Sciences, Nanjing University, Nanjing 210023, China; Department of Earth and Environmental Sciences, Tulane University, New Orleans, LA 70118, USA; Department of Civil and Environmental Engineering, Syracuse University, Syracuse, NY 13244, USA; State Key Laboratory of Environmental Geochemistry, Institute of Geochemistry, Chinese Academy of Sciences, Guiyang 550081, China; University of Chinese Academy of Sciences, Beijing 100049, China

**Keywords:** dissolved gaseous mercury, mercury stable isotope, atmospheric Hg deposition, re-emission, ocean mercury cycling

## Abstract

The sources and mechanisms driving fluxes of dissolved gaseous mercury (DGM) in aquatic ecosystems represent a critical yet poorly constrained component of the global mercury (Hg) cycle. Current models assume that DGM is primarily formed through the reduction of Hg^II^ in water, largely supplied by atmospheric Hg^II^ deposition. Here we quantify the Δ^200^Hg signatures of DGM across marine (median: 0.02‰) and freshwater (0.02‰) ecosystems, intermediate between water dissolved Hg^II^ and atmospheric Hg^0^. This indicates that DGM in natural waters is not derived solely from Hg^II^ reduction as previous assumed but also from atmospheric input of Hg^0^ during air-sea gas exchange. A Δ^200^Hg-based mixing model reveals that ∼40% and 54% of DGM in seawater and freshwater, respectively, is derived directly from atmospheric Hg^0^ input. Combining these findings with an existing oceanic Hg budget, we show that re-emission of previously deposited atmospheric Hg^0^ accounts for ∼70% of gross oceanic Hg^0^ evasion. Consequently, we demonstrate that existing models have systematically underestimated gross atmospheric Hg^0^ deposition while overestimating net oceanic Hg^0^ emissions.

## Teaser

This study shows the volatile dissolved gaseous mercury in nature waters is predominantly sourced from atmospheric elemental Hg input, suggesting that oceanic evasion represents a smaller source of atmospheric elemental Hg than previously recognized.

## INTRODUCTION

Mercury (Hg), particularly its organic forms (i.e. methylmercury species), are classified as neurotoxins with adverse effects on both wildlife and human populations [1]. Methylmercury, predominantly formed through microbial methylation of inorganic Hg in aquatic and terrestrial environments, can bioaccumulate to high levels in top-trophic fish and rice [2,3]. Atmospheric Hg^0^ and Hg^II^ deposition are the predominant Hg sources in aquatic ecosystems [4–6]. Following deposition, a large fraction (70%–89%) undergoes transformation to dissolve gaseous Hg (DGM, primarily Hg^0^) in surface waters in the presence of sunlight and dissolved organic matter and is subsequently re-emitted back to the atmosphere [7–10]. Current estimates of gross ocean Hg^0^ evasion (DGM evasion) are ∼4800 Mg yr^−1^, accounting for over half of global atmospheric Hg emissions [4–6,11,12]. The occurrence of DGM in seawaters therefore mitigates the formation of neurotoxic methylmercury mainly in the oxygen minimum zone by reducing the Hg burden in the seawater column and exerts a major influence on Hg cycling between the ocean and atmosphere.

A fundamental assumption in contemporary marine Hg biogeochemical models is that DGM production occurs primarily through photochemical and microbial reduction of Hg^II^ in natural waters (hereafter referred to water Hg^II^ reduction)—derived from atmospheric Hg^II^ deposition and from oxidation of atmospheric Hg^0^ following air-sea gas exchange [8]. In contrast, the contribution of direct atmospheric Hg^0^ input during air-sea gas exchange without subsequent oxidation (hereafter referred to direct atmospheric Hg^0^ input or deposition) to Hg^0^ evasion is considered negligible [8,11,13,14]. This prevailing view is mainly supported by the empirical parameterization of aqueous Hg^0^/Hg^II^ redox reactions and air-sea Hg^0^ exchange models, which holds that redox reaction rates are substantially faster than Hg^0^ gas exchange, suggesting atmospheric Hg^0^ deposition undergoes redox cycling rather than being promptly re-emitted [8,11]. Global models simulate bidirectional (reciprocal) air-sea Hg^0^ fluxes using a thin film gas exchange approach, estimating gross oceanic evasion and deposition fluxes at 4600–5000 Mg and 1700–2200 Mg yr^–1^, respectively, yielding a net ocean Hg^0^ efflux of ∼2800 Mg yr^–1^ [4–6].

Despite extensive DGM concentration measurements in marine and freshwater systems, significant uncertainties persist in quantifying air-sea Hg^0^ exchange flux due to incomplete understanding of Hg^0^ exchange mechanisms, parametrizations, and DGM sources [15]. Recent direct measurements of turbulent Hg^0^ fluxes over the coastal Baltic Sea revealed frequent net Hg^0^ deposition events (∼50% occurrence) even under conditions of oversaturation of DGM with respect to the solubility of atmospheric concentrations—a phenomenon inconsistent with thin film gas exchange model predictions [16]. Additionally, marine Hg isotope studies (primarily Hg^II^) suggest that modeled estimates of atmospheric Hg^0^ deposition to the ocean are likely to be significantly underestimated [11]. Note, these isotopic approaches only reflect the relative contributions of Hg^0^ and Hg^II^ to the small fraction of atmospheric Hg (11%–30%) that eventually accumulates in marine reservoirs (i.e. net deposition) [4–6,15,17], while the sources of most deposited Hg that is re-emitted to the atmosphere (i.e. oceanic DGM evasion) remain unclear. As a result, we lack the ability to resolve bidirectional Hg^0^ air-sea exchange fluxes, either individually or as a net composite net flux. Given that oceanic DGM evasion potentially represents the largest Hg emission flux to the atmosphere, quantifying this is critical for constraining bidirectional air-sea Hg^0^ exchange and ocean/atmosphere Hg chemical cycling. Nevertheless, this process remains poorly characterized.

Hg stable isotopes, particularly mass-independent fractionation of even-mass Hg isotopes (even-Hg MIF, Δ^200^Hg, and Δ^204^Hg signatures), can provide powerful tracers for tracking Hg sources and transformation pathways. Even-Hg MIF anomalies in the environment arise exclusively from high-altitude atmospheric photochemical reactions [18–20], resulting in characteristic negative Δ^200^Hg in atmospheric Hg^0^, and the reverse in atmospheric Hg^II^ samples [18–21]. If DGM production occurs predominantly through Hg^II^ reduction in natural waters as is conventionally assumed, DGM should exhibit an identical Δ^200^Hg signature as its aqueous Hg^II^ precursor.

**Figure 1.**
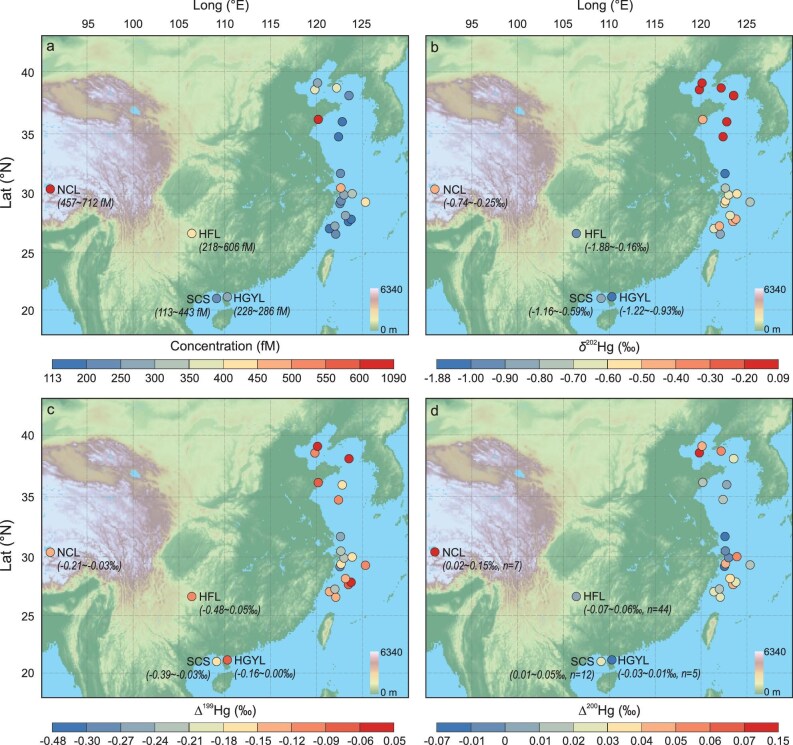
Spatial distribution patterns of DGM concentration (a) and corresponding δ^202^Hg (b), Δ^199^Hg (c), and Δ^200^Hg (d) signatures. In NCL, HFL, HGYL, and SCS, DGM were collected at fixed sampling sites and the concentrations and isotopic compositions shown in the figures are the median values (range) of all observations. In BHS, YS, and ECS, DGM were collected at stations along the cruise route.

To test this hypothesis, we present an investigation of the isotopic composition of ultra-trace-level DGM across diverse marine and freshwater ecosystems (Fig. 1). By coupling these measurements with concurrent analyses of water dissolved Hg (DHg) and atmospheric Hg^0^ isotopes, we quantify the relative contributions of water Hg^II^ reduction and direct atmospheric Hg^0^ input to DGM. Furthermore, by integrating the constrained DGM sources in this study and seawater Hg^II^ sources in a previous study [11], together with current best estimates of the global surface ocean Hg budget [4–6,15,17], we estimate the ratio of atmospheric Hg^0^ to Hg^II^ gross deposition to the ocean and the net oceanic Hg^0^ emissions.

## RESULTS AND DISCUSSION

### DGM concentration and isotope composition

DGM concentrations showed a large variation across marine and freshwater ecosystems. Higher DGM concentrations in seawaters were typically observed close to land (Fig. 1a). The median DGM concentrations in the Bohai Sea (BHS), Yellow Sea (YS), East China Sea (ECS), and South China Sea (SCS) seawaters and Hongfeng Lake (HFL), Huguangyan Lake (HGYL), and Nam Co Lake (NCL) freshwaters were 343, 605, 246, 239, 438, 264, and 647 fM, respectively (Fig. 1a, and Fig. S1a and Table S1). The overall median seawater DGM concentration was 271 fM (interquartile range (IQR): 198–389, *n* = 37), slightly lower than that in freshwaters (median: 440 fM, IQR: 362–499, *n* = 56). The DGM concentrations in seawater measured in this study align with previous observations in YS (median: 317 fM) [22], SCS (161 fM) [23], and the Mediterranean Sea (174 fM) [24], but were higher than those reported for the central Pacific (median: 63 fM) [25,26] and Atlantic Ocean (62 fM) [27].

**Figure 2.**
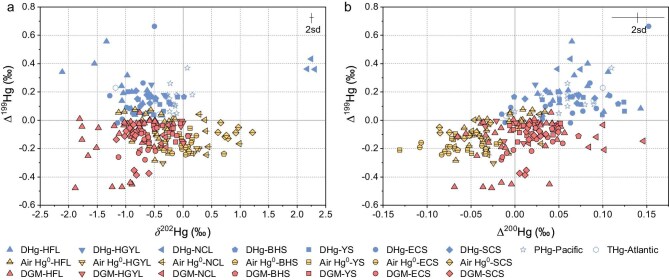
Scatterplots of isotopic compositions (∆^199^Hg vs δ^202^Hg (a) and Δ^200^Hg (b)) of DGM and DHg in water, as well as Hg^0^ in the surrounding ambient air. PHg in the Pacific Ocean (PHg-Pacific) and THg in the Atlantic Ocean (THg-Atlantic) are from Jiskra *et al.* [11], while other values were made for this study. The representative analytical uncertainty at ±2σ is established through repeated measurements of an international standard reference (NIST RM 8610).

DGM stable isotopes in seawater and freshwater exhibited negative δ^202^Hg and Δ^199^Hg signatures, with near-zero Δ^200^Hg signatures (Fig. 2). The median (IQR) DGM Δ^200^Hg values were 0.06‰ (0.05 to 0.07‰, *n* = 2), 0.01‰ (0.00 to 0.03‰, *n* = 10), 0.02‰ (0.00 to 0.04‰, *n* = 13), 0.02‰ (0.01 to 0.03‰, *n* = 12), 0.02‰ (−0.02 to 0.04‰, *n* = 44), −0.01‰ (−0.02 to 0.01‰, *n* = 5), and 0.09‰ (0.06 to 0.10‰, *n* = 7) in BHS, YS, ECS, SCS, HFL, HGYL, and NCL, respectively (Fig. S2 and Table S1). DGM Δ^199^Hg displayed consistently negative values in both seawater and freshwater, with the median (IQR) values of −0.09‰ (−0.10 to −0.07‰), −0.08‰ (−0.10 to −0.04‰), −0.15‰ (−0.22 to −0.12‰), −0.10‰ (−0.19 to −0.08‰), −0.05‰ (−0.14 to −0.02‰), −0.04‰ (−0.15 to −0.02‰), and −0.15‰ (−0.18 to −0.11‰) in BHS, YS, ECS, SCS, HFL, HGYL, and NCL, respectively. The median DGM δ^202^Hg values ranged from −0.87 to −0.05‰ in the BHS, YS, ECS, and SCS seas and from −0.97 to −0.35‰ in the HFL, HGYL, and NCL lakes. Seawater DGM δ^202^Hg values increased with latitude, while DGM concentrations, Δ^199^Hg, and Δ^200^Hg showed no clear spatial trends with latitude (Fig. S3).

### Water DHg and atmospheric Hg^0^ concentration and isotope composition

The elevated concentrations of DGM in this study were concomitant with heightened water DHg and atmospheric Hg^0^ levels (Fig. S1b and c). The median (IQR) water DHg concentrations in the seas and lakes investigated were 1.69 (1.19–2.52) and 10.27 (7.99–12.70) pM, respectively (Table S1). These values were ∼1.7 and 10 times higher than the median global surface seawater (e.g. 0.5–1.3 pM) [11,26,28,29]. The median atmospheric Hg^0^ concentrations in BHS (2.50 ng m^−3^, *n* = 2), YS (2.40 ng m^−3^, *n* = 8), ECS (2.02 ng m^−3^, *n* = 12), and HFL (4.40 ng m^−3^, *n* = 26) exceeded the Northern Hemispheric background in 2014 (1.51 ng m^−3^) [30]. Conversely, the median concentrations in SCS (1.13 ng m^−3^, *n* = 8), HGYL (1.11 ng m^−3^, *n* = 6), and NCL (1.16 ng m^−3^, *n* = 6) were slightly lower than the Northern Hemispheric background (Table S1).

Seawater DHg samples (*n* = 32) had a median (IQR) δ^202^Hg of −0.69‰ (−0.83 to −0.49), a median (IQR) Δ^199^Hg of 0.12‰ (0.06 to 0.16), and a median Δ^200^Hg of 0.07‰ (0.03 to 0.09). Freshwater DHg samples (*n* = 22) showed median (IQR) δ^202^Hg, Δ^199^Hg, and Δ^200^Hg of −0.67‰ (−1.03 to −0.41), 0.20‰ (0.17 to 0.33), and 0.06‰ (0.04 to 0.08), respectively (Fig. 2 and Table S1). These values align with previous observations of total mercury (THg) in the Atlantic Ocean and Mediterranean Sea [11]. Atmospheric Hg^0^ exhibited median (IQR) δ^202^Hg of 0.10 (−0.02 to 0.65) over the seas and −0.31 (−0.49 to 0.05) over the lakes, with corresponding median (IQR) Δ^199^Hg of −0.16‰ (−0.21 to −0.11) and −0.09‰ (−0.19 to 0.00), and median (IQR) Δ^200^Hg of −0.06‰ (−0.08 to −0.04) and −0.03‰ (−0.04 to −0.01), respectively (Fig. 2 and Table S1).

DGM Δ^199^Hg values were consistently more negative compared to DHg across all seas and lakes (median Δ^199^Hg_(DGM-DHg)_ of −0.23‰ in seawater and −0.35‰ in freshwater, Fig. S2b), suggesting that photoreduction of Hg^II^ complexed with oxygen-containing ligands, which imparts negative Δ^199^Hg signatures to the Hg^0^ product, as the major driver. In contrast, microbial and abiotic dark reduction, as well as photoreduction of Hg^II^ bound to other organic ligands (e.g. nitrogen, sulfur, and carbon-containing), would yield Δ^199^Hg similar to or more positive than those of DHg (Fig. S4) [31–33]. Moreover, the absence of clear diel variations in DGM Δ^199^Hg suggests that microbial and abiotic dark reduction are weak (Fig. S5), otherwise a positive shift in DGM Δ^199^Hg at night would be expected. Atmospheric input of Hg^0^ may also contribute to the observed negative DGM Δ^199^Hg. Although oxidation of Hg^0^ in water could impart a minor positive Δ^199^Hg shift [34], it may be insufficient to counterbalance the initial negative signature of the atmospheric Hg^0^, resulting in the overall negative Δ^199^Hg in the remaining aqueous Hg^0^ directly deposited from the atmosphere (Fig. S6).

### Gross Hg^0^ evasion flux and isotope composition

Gross Hg^0^ evasion fluxes across the investigated seas ranged from 1.1 to 34.4 ng m^−2^ h^−1^ (*n* = 34), with median (IQR) values of 2.6 (1.9–3.3), 3.9 (2.5–6.7), 7.3 (2.4–11.8), and 7.9 (6.2–9.0) ng m^−2^ h^−1^ in YS, BHS, ECS, and SCS, respectively. These values are comparable to those previously reported for coastal oceans globally, with a median of 1.4–7.0 ng m^−2^ h^−1^ (*n* = 6, Table S2). Combining ours with global observations, the median gross Hg^0^ evasion flux in coastal oceans is 4.6 ng m^–2^ h^–1^ (IQR: 2.4–8.2, *n* = 277, Fig. S7), ∼3.3 times higher than the median value for global open oceans (∼1.4 ng m^−2^ h^−1^) [11]. Based on the measured fluxes and corresponding sea surface areas (see Text S5 and Table S2), we estimate that coastal oceans emit 1155 (711–2118) Mg yr^−1^ of gross Hg^0^. Although covering only 7.2% of the global ocean surface, coastal oceans contribute nearly one-quarter of the global gross oceanic Hg^0^ evasions [4,5,11,35]. Despite this significance, our estimate remains subject to significant uncertainty due to limited field observations in these regions (Fig. S7).

Evasion of DGM from waters, primarily seawaters, represents an important source of atmospheric Hg^0^ [5,36,37]. Our observations indicate that Hg^0^ evasion from both seawater and freshwater exhibits negative δ^202^Hg and Δ^199^Hg and near zero Δ^200^Hg signatures. Given the MDF fractionation factor (ε^202^Hg_air-water_) of −0.44‰ and negligible odd-Hg MIF obtained from DGM volatilization experiments [38], we estimate that the median δ^202^Hg, Δ^199^Hg, and Δ^200^Hg of oceanic gross Hg^0^ evasions are −1.04‰ (IQR: −1.29 to −0.89), −0.11‰ (IQR: −0.15 to −0.07), and 0.02‰ (IQR: 0.01 to 0.04), respectively. These values are similar to soil Hg^0^ emissions (δ^202^Hg: −1.13‰, Δ^199^Hg: −0.13‰, Δ^200^Hg: 0.02‰) [39,40], but are clearly distinct from anthropogenic Hg^0^ sources, characterized by a median δ^202^Hg of −0.58‰ and near-zero Δ^199^Hg and Δ^200^Hg [41].

### DGM sources from water and the atmosphere

**Figure 3.**
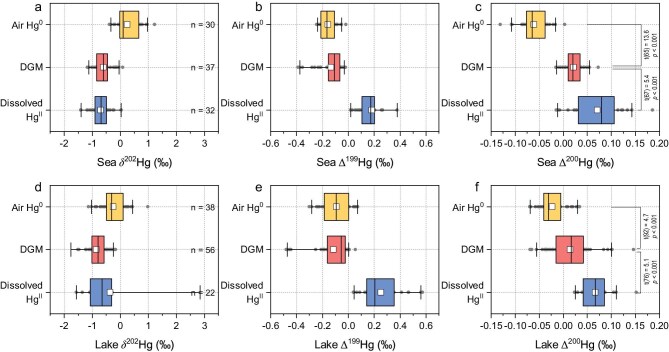
Comparison of the isotopic compositions of Hg^0^ in ambient air and DGM and dissolved Hg^II^ in waters. Panels (a), (b), and (c) illustrate the δ^202^Hg, Δ^199^Hg, and Δ^200^Hg signatures found in marine environments, while panels (d), (e), and (f) depict these signatures in freshwater lakes. The isotopic compositions of dissolved Hg^II^ are derived by subtracting the isotopic compositions of total dissolved mercury (DHg) from those of DGM (see Text S7). The white square and black line within the box represent the mean and median values, respectively. The boxes, whiskers, and gray dots indicate the interquartile range (IQR), the 5th to 95th percentile range, and individual data points outside the IQR, respectively. The Δ^200^Hg values of air Hg^0^, DGM, and dissolved Hg^II^ in marine and lake environments did not significantly deviate from normality (K-S test, *p* = 0.09 to 0.17). The statistical difference is tested using the Independent-Sample T test method.

Current understanding of Hg biogeochemical cycling in the surface ocean indicates that DGM originates primarily from *in situ* reduction of Hg^II^ and, to a lesser extent, from direct atmospheric Hg^0^ input (subsequently unoxidized) [14]. Nevertheless, the global production of DGM through photochemical and biological Hg^II^ reduction is estimated at 206 × 10^3^ Mg yr^–1^ [8], ∼100 times higher than gross atmospheric Hg^0^ deposition flux (1700 to 2200 Mg yr^−1^) [4,5,17]. Furthermore, atmospheric Hg^0^ dissolved into surface waters is thought to be rapidly oxidized [13,42] and largely converted to Hg^II^ before potential re-emission. Therefore, current thinking is that DGM in natural waters is predominantly derived from Hg^II^ reduction in water, with negligible contributions from direct atmospheric Hg^0^ input. However, in our study DGM Δ^200^Hg were significantly lower than those of dissolved Hg^II^ in both seawater and freshwater samples (Independent-Sample T test, t(67) = −5.4 and *p *< 0.001 in seawater; t(76) = −5.1 and *p *< 0.001 in freshwater). Additionally, both DGM and dissolved Hg^II^ Δ^200^Hg were greater than atmospheric Hg^0^ (Independent-Sample T test, *p *< 0.001 for all, Fig. 3c and f and Fig. S2c). These observations suggest that DGM production in natural waters cannot be explained solely by water Hg^II^ reduction, but rather requires substantial contribution from direct atmospheric Hg^0^ deposition that is not subsequently oxidized.

We employed a binary Δ^200^Hg mixing model to estimate the relative contributions of direct atmospheric Hg^0^ input and *in situ* Hg^II^ reduction to DGM in both seawaters and freshwaters (see Text S8). Measured Δ^200^Hg values of dissolved Hg^II^ and atmospheric Hg^0^ from diverse marine and lacustrine environments were used as endmembers representing these two sources. The production of DGM via Hg^II^ reduction in natural waters is predominantly linked to the dissolved Hg pool [8,42–44]. Our findings (Fig. S8) show that direct atmospheric Hg^0^ deposition that is not subsequently oxidized contributes a median of 40% (IQR: 35%–54%, *n* = 37) of DGM in seawaters and 54% (median; IQR: 42%–74%, *n* = 56) in freshwaters. In marine environments, the highest contributions from direct atmospheric Hg^0^ input were observed in the Yellow Sea (median: 57%, IQR: 48%–61%, *n* = 10), followed by the East China Sea (median: 42%, IQR: 37%–54%, *n* = 13) and the South China Sea (median: 38%, IQR: 32%–40%, *n* = 12), with the lowest values in the Bohai Sea (median: 28%, IQR: 25%–31%, *n* = 2). In freshwater lakes, the greatest contributions were found in HGYL (median: 63%, IQR: 57%–67%, *n* = 5), followed by HFL (median: 56%, IQR: 47%–73%, *n* = 44) and NCL (median: 23%, IQR: 20%–33%, *n* = 7).

Our findings suggest that atmospherically deposited Hg^0^ is not effectively oxidized to Hg^II^ in surface waters. Rather following deposition, a substantial fraction persists as aqueous Hg^0^ (DGM) and is subsequently re-emitted back to the atmosphere. This mechanism is in contrast with the current understanding of Hg biogeochemical cycling in the surface ocean [8,11]. The relative contribution of direct atmospheric Hg^0^ input to DGM depends on both deposition flux and *in situ* Hg^0^/Hg^II^ redox dynamics. Hg redox reactions occur at a slower rate in freshwater compared to seawater [44,45], which may explain the higher contributions of direct atmospheric Hg^0^ input to DGM in the lakes studied. Utilizing the methodology of Jiskra *et al.* (see Text S4) [11], we estimated gross atmospheric Hg^0^ deposition fluxes across the seas studied with a range from 0.1 to 2.9 ng m^−2^ h^−1^ (median: 0.5). These values are substantially lower than previously reported photooxidation flux (60.0 ng m^−2^ h^−1^) [13] and our estimated mean DGM production flux (69.0 ng m^−2^ h^−1^) from water-column Hg^II^ photoreduction, using a photoreduction rate of 0.32 h^−1^ and assuming a reducible dissolved Hg^II^ fraction of 40% in 1.8 m of seawater [8,42]. Therefore we propose that the bidirectional water-air exchange of Hg^0^ may occur at a substantially faster rate than previously assumed due to our incomplete understanding of the parameterization of the Hg^0^ transfer velocity [16], or that the kinetic rate constants of redox reactions have been overestimated. A higher exchange rate would facilitate rapid atmospheric Hg^0^ invasion and swift DGM evasion, implying a short residence time for Hg^0^ in aquatic ecosystems. Although this rapid bidirectional exchange may have limited impact on the net gas exchange flux, it could rival rapid aqueous redox processes, allowing a notable fraction of DGM to originate directly from atmospheric Hg^0^. In aqueous systems, Hg^0^ oxidation and Hg^II^ reduction are predominately photochemically driven [13,42], yet reported rate constants vary largely by up to 1∼2 orders of magnitude [8,46]. Overall, our findings suggest that atmospheric Hg^0^ deposition fluxes should be of a similar order of magnitude as in-water redox transformation.

### Implications for Hg cycling between the atmosphere and ocean

**Figure 4.**
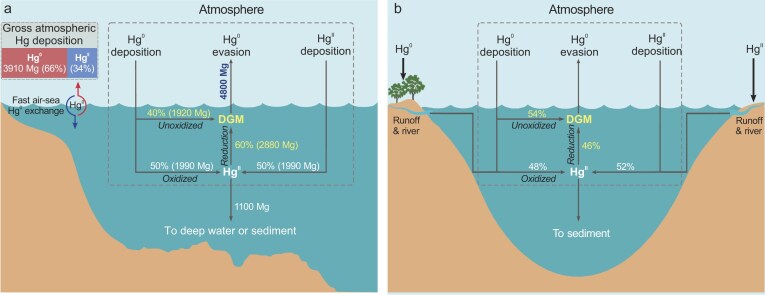
Updated atmospheric Hg deposition and re-emission in natural waters, as derived from the constrained DGM sources in this study. Panels (a) and (b) represent seawater and freshwater, respectively. We observe that 40% of DGM in seawater and 54% in freshwater is sourced from direct Hg^0^ atmospheric deposition _(subsequently unoxidized)_. The remaining portions are generated through the reduction of mercuric ions (Hg^II^), which are supplied by atmospheric Hg^0^ deposition _(subsequently oxidized)_ and Hg^II^ deposition at ratios of 1:1 for seawater (Jiskra *et al.*, 2021) [11] and 0.94:1 for freshwater (this study). The total contribution of atmospheric Hg^0^ to DGM, both directly and indirectly (to Hg^II^ followed by aqueous reduction), accounts for 70% in seawater (a) and 76% in freshwater (b). The gross deposition fluxes of atmospheric Hg^0^ and Hg^II^ are estimated based on model calculations of the gross oceanic Hg^0^ evasion flux (∼4800 Mg yr^−1^), alongside a net atmospheric Hg sink in the ocean (1100 Mg yr^−1^) [5].

Our data collected from diverse marine ecosystems indicate consistent contributions of direct atmospheric Hg^0^ input to DGM regardless of latitude, vertical water depth, and proximity to land (*p *> 0.05 for all, ANOVA, Fig. S9). Furthermore, the contributions of direct atmospheric Hg^0^ input to DGM under low atmospheric Hg^0^ (e.g. ≤1.50 ng m^−3^) and seawater DHg (e.g. ≤1.30 pM) conditions, typical of open ocean settings, were comparable to those under elevated atmospheric Hg^0^ and seawater DHg levels (Fig. S10). This pattern suggests that a substantial portion of DGM from direct atmospheric Hg^0^ input is likely a widespread occurrence in ocean environments. Given that the atmospheric Hg^0^ deposition also accounts for ∼50% (median, IQR: 34%–67%) of Hg^II^ in seawater globally [11], we estimate that the total contribution of atmospheric Hg^0^ deposition via direct input and Hg redox-cycling is 70% (median, IQR: 57%–85%) of seawater DGM pool (Fig. 4a). For freshwater ecosystems the contribution is slightly higher at 76% (median, IQR: 64%–89%) (Fig. 4b). These findings indicate that the evasion of Hg^0^ from natural waters is predominantly driven by the re-emission of atmospherically deposited Hg^0^.

Atmospheric Hg^0^ and Hg^II^ deposition to surface ocean is balanced by re-emission of Hg^0^ to the atmosphere (DGM evasion) and transport of Hg^II^ to deep water or sediment (sedimentation). We couple the latest model estimates by Shah *et al.* [5] of a gross ocean Hg^0^ evasion flux (4800 Mg yr^−1^) and net transport Hg^II^ from the surface ocean to deep water or sediment (1100 Mg yr^−1^), together with our observations of the relative contributions of atmospheric Hg^0^ to DGM and Hg^II^ in seawater [11] to estimate gross atmospheric Hg^0^ deposition. This result shows a gross atmospheric Hg^0^ deposition flux of 3910 Mg yr^−1^ to the oceans globally representing 66% of total atmospheric Hg deposition and a Hg^0^:Hg^II^ deposition ratio of ∼2:1 (Fig. 4a). We also made estimates based on the Hg evasion and sedimentation flux data from other oceanic models [4,6,15,17], that show similar gross atmospheric Hg^0^ deposition contributions of 64%–68% (Table S3).

The estimated contribution of atmospheric gross Hg^0^ deposition to the ocean, derived from the DGM Δ^200^Hg mixing model, exceeds earlier modeling results (27%–40%) [4–6,15,17] and the marine sample Δ^200^Hg-based estimate (50%) by Jiskra *et al.* [11]. The possible reasons for the low contribution of atmospheric gross Hg^0^ deposition in global ocean models have been speculated by Jiskra *et al.* [11], such as overestimate of dry and wet Hg^II^ deposition or incomplete understanding of the air-sea Hg^0^ exchange parameterizations. Estimates based on marine Δ^200^Hg samples only reflect the contribution of atmospheric Hg^0^ to the dissolved and particulate Hg^II^ pool [11], without considering atmospheric Hg^0^ that has re-evaded back to the atmosphere, and therefore represent the net, rather than the gross, atmospheric Hg^0^ deposition to the ocean. Although Jiskra *et al.* incorporated DGM in their THg samples from seawater, the steady-state DGM pool represents merely ∼5% of annual DGM production in surface seawater [5,8,12], which is largely driven by atmospheric Hg^0^ deposition rather than water Hg^II^ reduction. As a result, their approach underestimates the gross Hg^0^ uptake by the ocean.

Based on the steady-state assumption for Δ^200^Hg of atmospheric Hg (i.e. emission Δ^200^Hg equals deposition Δ^200^Hg), our previous study proposed a potential bias towards Hg^II^ deposition pathways in global models [20]. Importantly, gross oceanic evasion of Hg^0^ constitutes the dominant global source of atmospheric Hg. In our earlier study, a mean Δ^200^Hg signature of 0.05‰ for gross oceanic Hg^0^ evasion was estimated based on seawater Hg^II^ observations [11]. However, this value appears high relative to DGM measurements reported here (median 0.02‰). Accordingly, we have revised the Δ^200^Hg signatures for various atmospheric Hg emission sources, incorporating both current and prior observations, yielding a median Δ^200^Hg of 0.01‰ (IQR: −0.01 to 0.03) for global gross Hg emissions (Table S4).

Assuming equivalence between emission and deposition Δ^200^Hg (see Text S9) and combining global Δ^200^Hg signatures for atmospheric Hg^0^ (median: −0.06‰, IQR: −0.08 to −0.04, *n* = 1120) with wet and dry Hg^II^ deposition (including precipitation, gaseous, and particulate Hg^II^; median: 0.15‰, IQR: 0.10 to 0.18, *n* = 898, Table S4 and Fig. S11), we estimate that atmospheric Hg^0^ deposition accounts for ∼66% (IQR: 60%–73%) of total atmospheric Hg deposition on a global scale. This estimate closely matches the contribution of Hg^0^ to the global ocean (66%, this study) and to individual land ecosystems (53%–80%) reported previously [47–49]. Overall, our findings indicate that atmospheric Hg^0^ deposition plays a more significant role in global Hg cycling than has been previously acknowledged.

Global air-sea Hg exchange models, based on water-air Hg^0^ gradient and exchange velocity parameterization, estimate net ocean Hg^0^ emissions at ∼2800 Mg yr^–1^ [8,12]. These models, however, did not consider the origins of DGM from direct atmospheric Hg^0^ input. Here we combine the atmospheric Hg^0^ contributions to DGM (this study) and Hg^II^ in seawater [11] as well as the gross ocean Hg^0^ evasion [5] to estimate the global net ocean Hg^0^ emissions at ∼890 Mg yr^−1^ (Fig. 4a and Table S3), ∼3-fold lower than earlier estimates. While preliminary, our results strongly suggest that the thin-film gas exchange model has underestimated global gross atmospheric Hg^0^ deposition and overestimated net oceanic Hg^0^ emissions. This discrepancy implies that ocean emissions may represent a smaller source of atmospheric Hg emissions globally than previously recognized, delivering new insights into recent declines in atmospheric Hg in the Northern Hemisphere and informing future projections on the recovery of Hg contamination under the Minamata Convention [50,51]. Additionally, our revised estimate of atmospheric Hg^0^ deposition implies a reduced net oxidation of atmospheric Hg^0^ (−1910 and −2577 Mg yr^−1^ over the ocean and globally) and a lower net Hg^II^ reduction to Hg^0^ in the surface ocean (−1920 Mg yr^−1^) than prior assumptions (Fig. S12) [5], the former of which is consistent with recent field-based findings that less Hg^II^ is deposited from the atmosphere to terrestrial and marine ecosystems than previously acknowledged [52, and references therein]. Recent computational and modelling studies have suggested that gas-phase oxidized Hg^I^ and Hg^II^ species would efficiently photodissociate back to Hg^0^ and offset atmospheric Hg^0^ oxidation [53,54], which may partly explain the lower net atmospheric Hg^0^ oxidation. Given the complex Hg redox chemistry and the numerous poorly-understood mechanisms and reaction rates [28,54,55], further research into the transport and transformation of Hg species in the atmosphere and ocean is critical for advancing a comprehensive understanding of the global Hg biogeochemical cycle.

## MATERIALS AND METHODS

DGM and DHg in natural waters, along with atmospheric Hg^0^, were simultaneously sampled during open research in the Bohai Sea, Yellow Sea, and East China Sea. *In situ* sampling was also conducted near Weizhou Island in the South China Sea, in the Hongfeng Lake in southwestern China, the Huguangyan Lake in southern China, and the high-altitude Nam Co Lake in the Tibetan plateau. Immediately after sampling, DGM in 120 L of seawater and 60 L of freshwater were extracted on to chlorine-impregnated carbon (CLC) traps following our previous study on board or at the sampling site [56]. DHg was initially filtered from 20 L of seawater and 10 L of freshwater and then pre-concentrated on to CLC traps [57]. Atmospheric Hg^0^ was collected using CLC traps [58]. DGM, DHg, and air Hg^0^ pre-concentrated in CLC traps were further pre-concentrated into 5 mL of mixed concentrated acid (2 HNO_3_:1 HCl, v/v). Hg isotope ratios were determined utilizing a Nu Plasma II (Nu Instrument Ltd., UK) multi-collector inductively coupled plasma mass spectrometer (MC-ICPMS). MDF Hg isotope composition (in delta notation, δ) is reported as referenced to the bracketing NIST SRM 3133 standard [59]: δ^xxx^Hg = [(^xxx^Hg/^198^Hg)_sample_/(^xxx^Hg/^198^Hg)_Nist 3133_–1], where ^xxx^Hg are ^199^Hg, ^200^Hg, ^201^Hg, ^202^Hg, and ^204^Hg, respectively. MIF signatures are expressed in capital delta (Δ) and calculated using the kinetic MDF law [59]: Δ^xxx^Hg = δ^xxx^Hg—(β^xxx^ × δ^202^Hg), where β^xxx^ is 0.252, 0.5024, 0.752, and 1.492 for ^199^Hg, ^200^Hg, ^201^Hg, and ^204^Hg, respectively. Relative contributions of direct atmospheric Hg^0^ input to DGM were calculated using a binary Δ^200^Hg mixing model: *f*_Hg0_ × Δ^200^Hg_Hg0_ + (1−*f*_Hg0_) × Δ^200^Hg_HgII_ = Δ^200^Hg_DGM_. The fraction of atmospheric Hg^0^ deposition in total atmospheric Hg deposition on a global scale is estimated using a binary Δ^200^Hg balance model: ∑*_l_*(Emis*_l_* × Δ^200^Hg*_l_*)/∑*_l_*Emis*_l_ *= *f*_Hg0_ × Δ^200^Hg_Hg0_ + (1− *f*_Hg0_) × Δ^200^Hg_HgII_. A Monte Carlo simulation was used to estimate the uncertainty of *f*_Hg0_. Detailed information of the open research cruise and *in situ* sampling sites, field sampling, sample processing and Hg pre-concentration, Hg concentration and isotope analysis, procedural blanks as well as air-sea Hg^0^ exchange flux, water dissolved Hg^II^ isotopic compositions, DGM Δ^200^Hg mixing model, and atmospheric Δ^200^Hg mass balance model are described in Text S1–S9.

## Supplementary Material

nwaf590_Supplemental_Files

## Data Availability

All data are available in the main text or Supplementary Data.
