# Supplementary material for: Atmospherically deposited elemental mercury drives evasion of mercury from the ocean and freshwaters
Source: Natl Sci Rev. 2025 Dec 26;13(2):nwaf590. doi: 10.1093/nsr/nwaf590 (PMC12831025; doi:10.1093/nsr/nwaf590)
Supplement: nwaf590_Supplemental_Files [file nwaf590_supplemental_files.zip › Supplementary Data.pdf]

Supplementary Data to:

Atmospherically deposited elemental mercury drives evasion of mercury from the ocean and freshwaters

Xuewu Fu,[1] Hui Zhang,[1] Kaihui Tang,[1,2] Jonas Sommar,[1] Jen-How Huang,[1] Zhengcheng Song,[3] Yanxu Zhang,[4] Charles T. Driscoll,[5] Xinbin Feng,[1,2*]

[1]State Key Laboratory of Environmental Geochemistry, Institute of Geochemistry, Chinese Academy of Sciences, Guiyang 550081, China

[2]University of Chinese Academy of Sciences, Beijing 100049, China

[3]School of Atmospheric Sciences, Nanjing University, Nanjing, 210023, China

[4]Department of Earth and Environmental Sciences, Tulane University, New Orleans, LA, 70118, USA

[5]Department of Civil and Environmental Engineering, Syracuse University, Syracuse, New York 13244, United States

*Corresponding author: Xinbin Feng, Email: fengxinbin@mail.gyig.ac.cn

Supplementary Data Text

Text S1……………………………………………………………………………Page S1
Text S2……………………………………………………………………………Page S1
Text S3……………………………………………………………………………Page S2
Text S4……………………………………………………………………………Page S2
Text S5……………………………………………………………………………Page S3
Text S6……………………………………………………………………………Page S3
Text S7……………………………………………………………………………Page S4
Text S8……………………………………………………………………………Page S4
Text S9……………………………………………………………………………Page S4

Supplementary Data Figures

Fig. S1……………………………………………………………………………Page S6
Fig. S2……………………………………………………………………………Page S7
Fig. S3……………………………………………………………………………Page S8
Fig. S4……………………………………………………………………………Page S9
Fig. S5……………………………………………………………………………Page S10
Fig. S6……………………………………………………………………………Page S11
Fig. S7……………………………………………………………………………Page S12
Fig. S8……………………………………………………………………………Page S13
Fig. S9……………………………………………………………………………Page S14
Fig. S10…………………………………………………………………………Page S15
Fig. S11…………………………………………………………………………Page S16
Fig. S12…………………………………………………………………………Page S17

Supplementary Data references

Supplementary Data Text

Text S1. Study area

Surface waters and atmospheric Hg^0 were sampled aboard the research vessel 'Lanhai 101' during the open research cruise NORC2023-01 in the Bohai Sea and Yellow Sea (July 13 - August 14 2023). Additional sampling were performed aboard RV 'Xiangyanghong 18' during the NORC2023-02 and NORC2023-301 cruises in the East China Sea (April 30 - May 7 2023). *In situ* sampling also took place near Weizhou Island in the South China Sea (109.104°E, 21.008°N; December 24 - 30 2022); at two sites in the Hongfeng Lake (106.408°E, 26.538°N and 106.419°E, 26.488°N, both 1150 m a.s.l.) during three periods in 2020 (January 5 - 13, 2020, May 25 - 30, and July 21 - 31); in the Huguangyan Lake (110.277°E, 21.143°N, 57 m a.s.l.) from August 11 - 15, 2020; and in the high-altitude Nam Co Lake (90.990°E, 30.398°N, 4718 m a.s.l.) from September 26 to October 1, 2020.

Text S2. Sampling and processing

Surface seawater and freshwater for DGM and DHg isotope analysis were collected using a 5-L acid-cleaned acrylic manual water sampler. DGM was extracted following a standard manual method previously documented [1, 2]. Following sampling, 10-L and 12-L aliquots of freshwater and seawater, respectively, were gradually transferred into a 14-L borosilicate glass bubbler. This bubbler was then purged with Hg-free air at 4-5 L min^{-1} for 1.5 h to extract DGM onto a large chlorine-impregnated carbon (CLC) trap containing 800 mg of CLC [3]. To ensure sufficient DGM for isotope analysis, multiple extractions were performed, processing 60-80 L of freshwater (10-L per extraction) and 120 L of seawater (12-L per extraction). Consequently, pre-concentration of each DGM isotope in freshwater and seawater required 9-12 hours for freshwater and 15 hours for seawater. During purging, the bubbler was covered with tinfoil to protect it from light.

Water samples for DHg isotope analysis were initially filtered immediately after collection using 90 mm quartz microfiber filters (Whatman) pre-heated at 500°C for 6 h. The procedure followed US-EPA method 1631 for DHg pre-concentration [4], conducted either on board or at the sampling site. In summary, filtered water was transferred into a 10-L amber borosilicate glass bottle, acidified with 5‰ double-distilled HCl (v/v) and treated with a 5‰ BrCl solution (v/v) to convert all Hg compounds to Hg^{II} over 12 h. Following digestion, 10 L of freshwater and 20 L of seawater (added in two steps) were placed in a 14-L borosilicate glass bubbler. A 1‰ $NH_2OH \cdot HCl$ (v/v) was introduced to neutralize any excess free halogens, followed by 5‰ $SnCl_2$ (v/v) to reduce Hg^{II}. The solution was then purged with Hg-free air at 4-5 L min^{-1} for 1.5 h to pre-concentrate DHg onto CLC traps [5].

Atmospheric total gaseous mercury (TGM= GEM + GOM) was collected from the front deck of the ship at ~8 m above sea level in the BHS, YS, and ECS, and from the top of a smaller vessel at ~3 meters above the water surface in HFL. In the SCS, NCL, and HGYL, samples were obtained from the nearest shoreline at 2 m above ground. Sampling employed small CLC trap containing 500 mg of CLC, operating at a flow rate of 3 L min^{-1} for 24 h in marine environments and 48 h in lake settings[6]. Airborne particles were removed using a 0.2 μm Teflon filter at the sampling inlet. Recent observations of atmospheric gaseous Hg^{II} (GOM) and reactive Hg^{II} (RM) in marine boundary layer using the new cation exchange membranes method showed typical mean concentrations of <35 pg m^{-3} [7-9], which on average accounts

for ~2% of the TGM concentrations measured in this study (Table S1). Currently, observations of GOM isotope composition in marine boundary layer are lacking. Using the mean GOM Δ^{200}Hg and Δ^{199}Hg of 0.15‰ and 0.44‰, respectively, in the free troposphere [10], a ~2% GOM contribution to TGM would therefore not likely shift the GEM Δ^{200}Hg and Δ^{199}Hg measurably (e.g., <0.003‰ and 0.009‰, respectively). We therefore suggest that GOM would not confound our GEM isotope observations and consider our observations as GEM isotope composition.

Following field sampling and pre-concentration, CLC traps containing DGM, DHg, and atmospheric Hg^0 samples were sealed with silicone stoppers and secured within three successive polyethylene bags. They were then stored at room temperature, either on-site or in the laboratory. In the laboratory, thermal desorption at 900 °C under a flow of Hg-free argon gas released Hg compounds as Hg^0, which were purged and pre-concentrated into 5 mL of mixed concentrated acid (2 HNO_3:1 HCl, v/v) [5]. Then, trapping bottles and impingers were rinsed three times with Milli-Q water, and the rinse water was incorporated into the acid solution. Final solutions were stored at 2-4 °C prior to Hg isotope analysis.

Text S3. Hg analysis, concentrations, and procedural blanks, recovery

Hg concentrations in the final trap solutions were determined using the cold vapor atomic fluorescence spectroscopy method (CVAFS, Tekran 2500, Tekran® Inc., Canada), calibrated with NIST SRM 3133 and spiked with 20-200 pg of Hg. Concentrations of water DGM and DHg, and atmospheric Hg^0, were calculated by dividing the total Hg mass in the final trap solutions by their respective sample volumes.

The acrylic manual water sampler were cleaned with 5% double distilled HNO_3, rinsed with Milli-Q water, and sealed in a plastic bag prior to use. Purging bubblers were cleaned using concentrated distilled HNO_3 at 120 °C on a hot plate, rinsed with Milli-Q water, and sealed with silicone stoppers and plastic bags. HCl used was of trace metal grade and double-distilled. Solutions of BrCl, $NH_2OH{\cdot}HCl$, and $SnCl_2$ were prepared following US-EPA method 1631 [4].

The mean blanks for the large and small CLC traps were 161±50 and 101±31 pg of Hg (n = 10), respectively. Procedural blanks for DGM, including contributions from field purging, CLC trap, thermal desorption, acid trap solution, and laboratory analysis, were determined by substituting 10-L natural waters with Milli-Q water and purging with Hg-free air gas for 16-24 hours, yielding a mean procedural blank of 282±147 pg (±1σ, n = 10), or ~7.1% of the total DGM pre-concentrated in the final trap solutions. DHg procedural blanks, assessed similarly to DGM but with the addition of BrCl, $NH_2OH{\cdot}HCl$, and $SnCl_2$ solutions, averaged 408±237 pg (±1σ, n = 9), accounting for ~5.5% of the DHg mass in the final trap solutions. CLC trapping efficiencies for Hg^0 were assessed using known amounts of Hg^0 injections, with a mean recovery of 109±12.2% (±1σ, n = 6). DGM and DHg recoveries from natural waters exceeded 95% in our recent studies [3, 5].

Text S4. Air-sea Hg^0 exchange fluxes

Gross ocean Hg^0 evasion ($F_{evasion}$, ng m^{-2} h^{-1}), gross atmospheric Hg^0 invasion ($F_{invasion}$, ng m^{-2} h^{-1}), and net air-sea Hg^0 exchange fluxes ($F_{net\ flux}$, ng m^{-2} h^{-1}) were calculated following Jiskra et al. [11]:

$$F_{evasion} = K_w \times C_{DGM} \quad (1)$$

$$F_{invasion} = K_w \times C_{Hg^0}/H \quad (2)$$

$$F_{net\ flux} = F_{evasion} - F_{invasion} \quad (3)$$

where K_w and H are the gas transfer velocity of Hg^0 at the water-air interface (m h^{-1}) and the Henry's Law constant (dimensionless). These parameters were calculated using the equations outlined by Jiskra et al. [11] , along with the sea surface temperature and wind speed measured in the present study (Table S11). C_{DGM} and C_{Hg^0} refer to the measured water DGM and atmospheric Hg^0 concentrations, respectively, in this research.

Text S5. Global coastal ocean gross Hg^0 evasion

Gross Hg^0 evasions from coastal oceans worldwide are estimated by multiplying the gross Hg^0 evasion flux by the corresponding coastal sea surface area in each coastal ocean using the following equation:

$$Evasion = \sum_{i=1}^{n}(F_i \times A_i) \quad (4)$$

where *Evasion* is the total gross Hg^0 evasions from global coastal oceans. F_i is the median gross Hg^0 evasion flux in coastal ocean i, A_i is the sea surface area of coastal ocean i.

Observations of gross Hg^0 evasion fluxes from coastal oceans are available in the Pacific Ocean, Atlantic Ocean, Southern Ocean, Mediterranean Sea, and Baltic Sea (Fig. S7 and Table S2). For other costal oceans without observational evasion flux data, the median value of the existing observations is adopted to estimate the gross Hg^0 evasions in these coastal oceans. IQR (interquartile range) of the gross Hg^0 evasions from global coastal oceans were calculated using the IQR of the observed evasion fluxes in each coastal ocean in equation (4).

Text S6. Hg isotope analysis

Hg isotope ratios were determined utilizing a Nu Plasma II (Nu Instrument Ltd., UK) multi-collector inductively coupled plasma mass spectrometer (MC-ICPMS) at the State Key Laboratory of Environmental Geochemistry, Guiyang, China [3, 5]. Throughout the analytical sessions, the MC-ICPMS exhibited a signal intensity ranging from 1.3 to 1.5 V for the ^{202}Hg isotope at a concentration of 1.0 ng mL^{-1} for both samples and standards. The lowest concentration of Hg detected in trap solutions was 0.36 ng mL^{-1}, which corresponded to a ^{202}Hg isotope signal of approximately 0.5 V. MDF Hg isotope compositions (in delta notation, δ) were calculated using the following equation [12]:

$$\delta^{xxx}Hg = \left[\left({}^{xxx}Hg/{}^{198}Hg\right)_{sample}/\left({}^{xxx}Hg/{}^{198}Hg\right)_{NIST\ 3133} - 1\right] \times 1000‰ \quad (5)$$

where ^{xxx}Hg are ^{199}Hg, ^{200}Hg, ^{201}Hg, ^{202}Hg, and ^{204}Hg, and NIST 3133 is the bracketing NIST SRM 3133 standard with Hg concentrations that align with those of the samples. MIF signatures are expressed in capital delta (Δ) and calculated using the kinetic MDF law[12]:

$$\Delta^{xxx}Hg\ (‰) = \delta^{xxx}Hg - \left(\beta^{xxx} \times \delta^{202}Hg\right) \quad (6)$$

where β^{xxx} is 0.252, 0.5024, 0.752, and 1.492 for ^{199}Hg, ^{200}Hg, ^{201}Hg, and ^{204}Hg, respectively.

The isotopic compositions of NIST SRM 8610 (n = 69) and SRM 1947 (n = 19), and CRM BCR 482 (n = 19) standards were measured periodically, yielding mean (±2σ) δ^{202}Hg, Δ^{199}Hg, and Δ^{200}Hg values of -0.52±0.07‰, -0.01±0.07‰, and -0.00±0.06‰, -1.59±0.11‰, -0.63±0.10‰, and 0.07±0.05‰, and 1.18±0.23‰, 5.14±0.11‰, and 0.10±0.06‰, respectively. These results are consistent with previously reported [13] and referenced values. The larger 2σ values obtained from the long-term analysis of the NIST SRM 8610 standard or its sample duplicates were utilized to represent the final analytical 2σ uncertainty of the Hg isotopic compositions in this investigation.

Text S7. Dissolved Hg^{II} isotopic compositions

The isotopic compositions of water dissolved Hg^{II} were calculated as the difference between those of DHg and DGM using the following equations:

$$\delta^{202}Hg_{Hg^{II}} = \left(\delta^{202}Hg_{DHg} - \delta^{202}Hg_{DGM} \times f_{DGM}\right)/(1 - f_{DGM}) \quad (7)$$

$$\Delta^{199}Hg_{Hg^{II}} = \left(\Delta^{199}Hg_{DHg} - \Delta^{199}Hg_{DGM} \times f_{DGM}\right)/(1 - f_{DGM}) \quad (8)$$

$$\Delta^{200}Hg_{Hg^{II}} = \left(\Delta^{200}Hg_{DHg} - \Delta^{200}Hg_{DGM} \times f_{DGM}\right)/(1 - f_{DGM}) \quad (9)$$

where $\delta^{202}Hg_{Hg^{II}}$, $\Delta^{199}Hg_{Hg^{II}}$, and $\Delta^{200}Hg_{Hg^{II}}$ are the water dissolved Hg^{II} isotopic compositions, respectively. $\delta^{202}Hg_{DHg}$, $\Delta^{199}Hg_{DHg}$, and $\Delta^{200}Hg_{DHg}$ are the measured isotopic compositions of DHg, respectively. $\delta^{202}Hg_{DGM}$, $\Delta^{199}Hg_{DGM}$, and $\Delta^{200}Hg_{DGM}$ are the measured isotopic compositions of DGM, respectively. f_{DGM} is the fraction the DGM in DHg.

Text S8. DGM Δ^{200}Hg mixing model

The relative contributions of unoxidized atmospheric Hg^0 invasion to water DGM were calculated using a binary Δ^{200}Hg mixing model as follows:

$$f_{Hg^0} \times \Delta^{200}Hg_{Hg^0} + \left(1 - f_{Hg^0}\right) \times \Delta^{200}Hg_{Hg^{II}} = \Delta^{200}Hg_{DGM} \quad (10)$$

where f_{Hg^0} is the fraction of DGM originates from direct atmospheric Hg^0 invasion (subsequently unoxidized). $\Delta^{200}Hg_{Hg^0}$ and $\Delta^{200}Hg_{Hg^{II}}$ correspond to the endmembers of atmospheric Hg^0 invasion and the reduction of water dissolved Hg^{II}, respectively, in terms of Δ^{200}Hg. $\Delta^{200}Hg_{DGM}$ is the measured DGM Δ^{200}Hg. The production of DGM via Hg^{II} reduction in natural waters is predominantly linked to the dissolved Hg pool [14-17]. Note not all forms of dissolved Hg^{II} are reducible and the rapid re-equilibration between reducible and non-reducible forms is unlikely to generate even-Hg MIF. To assess the contribution of unoxidized Hg^0_{atmos} invasion and the associated uncertainty, we employed a Monte Carlo simulation. For each sea and lake, we utilized 10,000 sets of Δ^{200}Hg values that were randomly selected from the IQR of the endmembers, as well as from the mean - 2σ and the mean + 2σ of the DGM samples obtained in this study, to estimate the contributions from unoxidized Hg^0_{atmos} invasion.

Text S9. Atmospheric Δ^{200}Hg mass balance model

In global atmospheric Hg mass and isotope models, it is assumed that the flux and isotopic compositions of combined atmospheric deposition are counterbalanced by those of combined atmospheric emissions under steady-state conditions [10, 18, 19]. Consequently, we utilize the equivalent

emission and deposition Δ^{200}Hg to calculate the proportion of Hg^0 deposition within the overall atmospheric Hg deposition:

$$\sum_l (Emis_l \times \Delta^{200}Hg_l)/\sum_l Emis_l = f_{Hg^0} \times \Delta^{200}Hg_{Hg^0} + (1 - f_{Hg^0}) \times \Delta^{200}Hg_{Hg^{II}} \quad (11)$$

$$\Delta^{200}Hg_{Hg^0} = \sum_j (Dep_{Hg^0-j} \times \Delta^{200}Hg_{Hg^0-j})/\sum_j Dep_{Hg^0-j} \quad (12)$$

$$\Delta^{200}Hg_{Hg^{II}} = \sum_j (Dep_{wet\ Hg_j^{II}} \times \Delta^{200}Hg_{wet\ Hg_j^{II}} + Dep_{gas\ Hg_j^{II}} \times \Delta^{200}Hg_{gas\ Hg_j^{II}} + Dep_{particulte\ Hg_j^{II}} \times \Delta^{200}Hg_{particulate\ Hg_j^{II}}) / \sum_j (Dep_{wet\ Hg_j^{II}} + Dep_{gas\ Hg_j^{II}} + Dep_{particulte\ Hg_j^{II}}) \quad (13)$$

where $Emis_l$ and $\Delta^{200}Hg_l$ denote the emission flux and Δ^{200}Hg of source sector l, respectively. f_{Hg^0} indicates the contribution from atmospheric Hg^0 deposition. The terms $\Delta^{200}Hg_{Hg^0}$ and $\Delta^{200}Hg_{Hg^{II}}$ signify the combined Δ^{200}Hg signatures of flux-weighted atmospheric Hg^0 and Hg^{II} deposition, which includes wet, gaseous and particulate Hg^{II} deposition endmembers, respectively. The Δ^{200}Hg endmembers for atmospheric Hg^0 and Hg^{II} deposition in equations (12) and (13) were derived from the latitudinal variations in deposition flux and the Δ^{200}Hg values of atmospheric Hg forms (Fig. S11). Dep_{Hg^0-j} and $\Delta^{200}Hg_{Hg^0-j}$ represent the atmospheric Hg^0 deposition flux and mean Δ^{200}Hg within 10° latitude bins j, respectively. $Dep_{wet\ Hg_j^{II}}$, $Dep_{gas\ Hg_j^{II}}$, and $Dep_{particulte\ Hg_j^{II}}$ correspond to the deposition fluxes of precipitation, gaseous, and particulate Hg^{II} within the same 10° latitude bins j, respectively. Similarly, $\Delta^{200}Hg_{wet\ Hg_j^{II}}$, $\Delta^{200}Hg_{gas\ Hg_j^{II}}$, and $\Delta^{200}Hg_{particulate\ Hg_j^{II}}$ represent the mean Δ^{200}Hg of precipitation, gaseous, and particulate Hg^{II} in those latitude bins, respectively. In instances where Δ^{200}Hg observations are absent for certain latitudinal bins, the median and IQR from adjacent bins were utilized for calculations. The Δ^{200}Hg data for atmospheric Hg in the southern polar regions is sparse, necessitating the use of observations from the northern polar regions. Data regarding Δ^{200}Hg from various emission source sectors and speciation of atmospheric Hg are sourced from the literature (Table S4). The latitudinal deposition fluxes of speciated atmospheric Hg at intervals of 10° are derived from the work of Shah et al. [18]. In equation (11), a Monte Carlo simulation was conducted using 10,000 groups of Δ^{200}Hg values, which were randomly selected from the IQR of the estimated atmospheric emissions, as well as the deposition endmembers for atmospheric Hg^0 and Hg^{II}. This approach was utilized to estimate the contribution and uncertainty associated with atmospheric Hg^0 deposition (Table S4).

Supplementary Data Figures

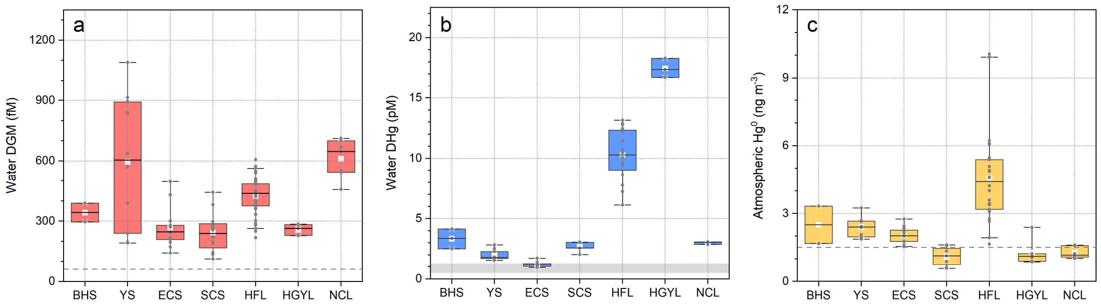

Fig. S1. Statistics of Hg concentrations in the seas and lakes. (a), (b), and (c) water DGM, water DHg, and atmospheric Hg^0, respectively. The white square and black line in the box are the mean and median, respectively. The boxes, whiskers, and gray dots represent the IQR, 5th-95th range, and individual values out of the IQR, respectively. The dash lines and gray color shaded area indicate the global DGM and DHg concentrations in open oceans[11, 17] and the mean atmospheric Hg^0 concentrations in the Northern Hemisphere [20].

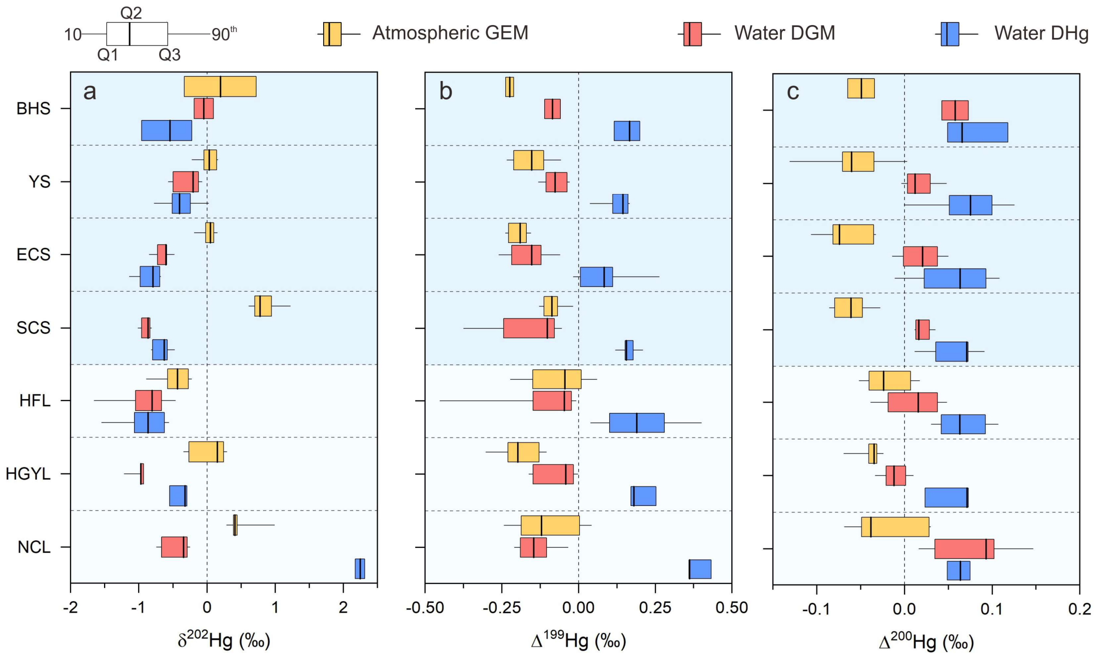

Fig. S2. Hg stable isotope in each sea and lake. (a), (b), and (c) δ^{202}Hg, Δ^{199}Hg, and Δ^{200}Hg, respectively. Δ^{199}Hg and Δ^{200}Hg of DGM almost fell in between water DHg and atmospheric Hg^0 in each sea and lake.

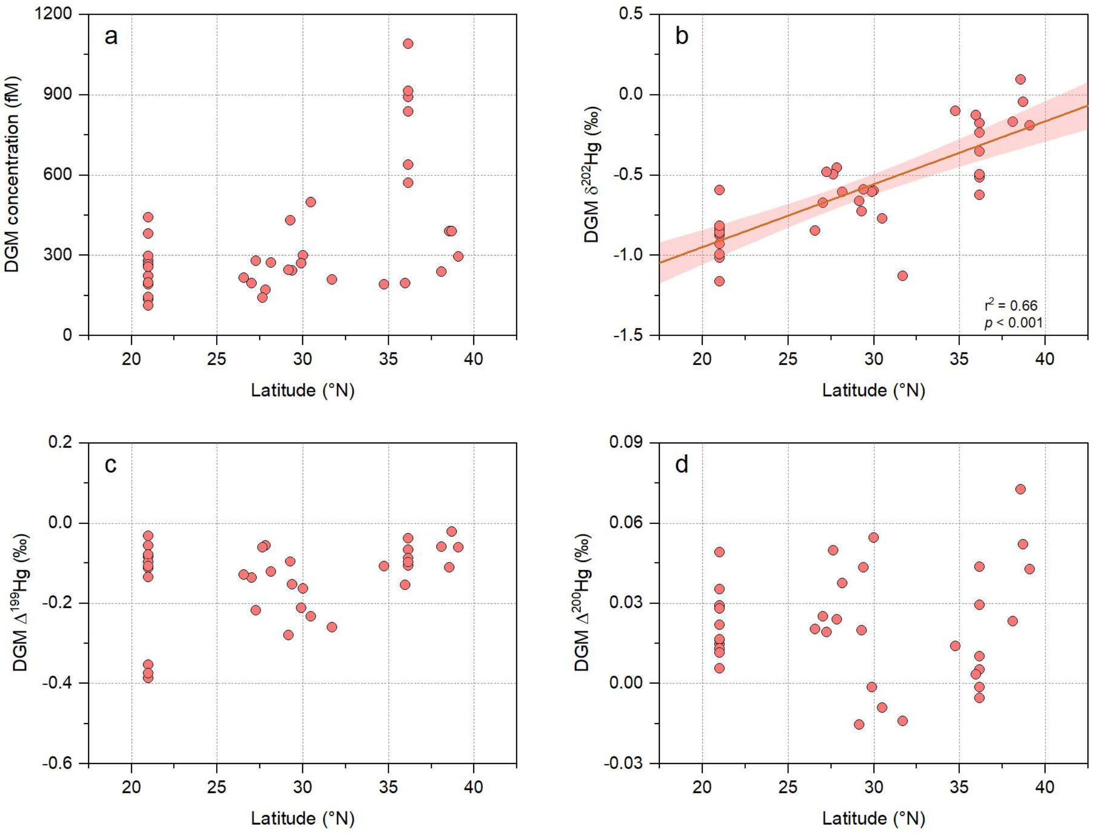

Fig. S3. Relationship between the Latitude and DGM concentration and isotope. (a), (b), (c), and (d) concentration, δ^{202}Hg, Δ^{199}Hg, and Δ^{200}Hg, respectively. DGM δ^{202}Hg were significantly positively correlated with latitude, and no significant correlations exist between the latitude and DGM concentration, Δ^{199}Hg, and Δ^{200}Hg (ANOVA, $p > 0.05$ for all).

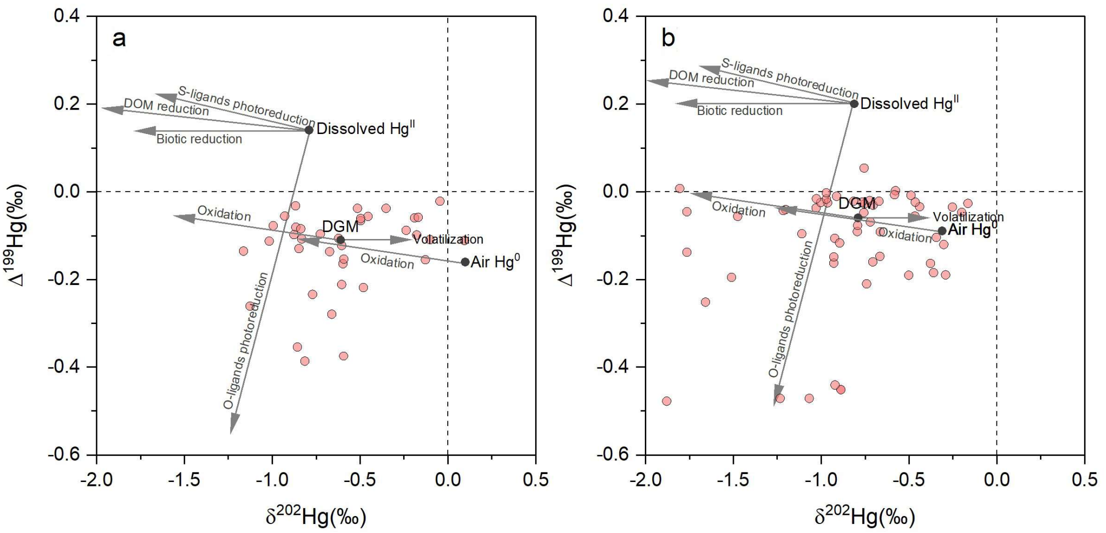

Fig. S4. DGM δ^{202}Hg and Δ^{199}Hg in natural waters and underling MDF and odd-Hg MIF mechanisms. (a) and (b) seawater and freshwater, respectively. Orange dots are the DGM isotopic compositions measured in this study. Black dots represent the median δ^{202}Hg and Δ^{199}Hg values of water dissolved Hg^{II} and DGM and atmospheric Hg^0 isotopic compositions over the seas and lakes. The fractionation trajectories of Hg isotopes during photochemical reduction of Hg^{II} complexed with S- and O-ligands, biotic and DOM Hg^{II} reduction, DGM oxidation and evaporation, and atmospheric Hg^0 oxidation followed by invasion are from literature [21-25].

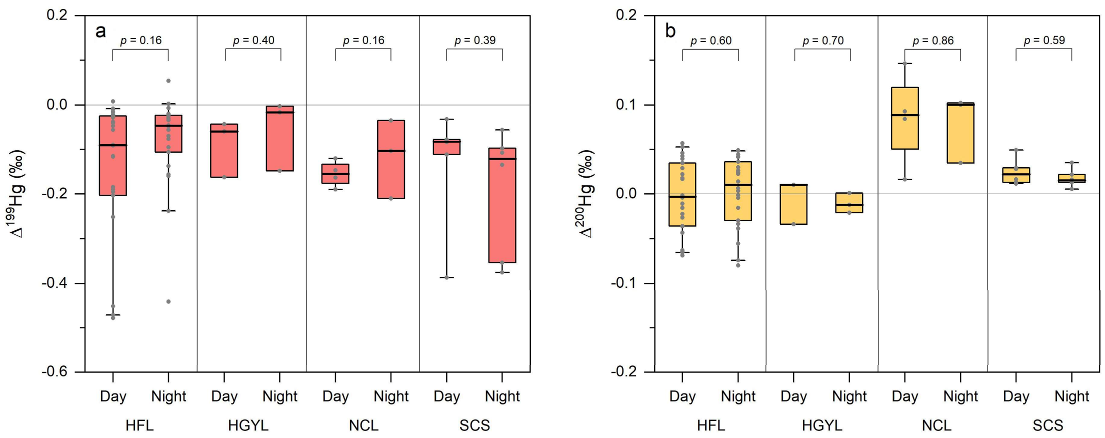

Fig. S5. Diurnal variations in DGM Δ^{199}Hg and Δ^{200}Hg. (a) and (b) Δ^{199}Hg and Δ^{200}Hg, respectively. No significant difference in Δ^{199}Hg and Δ^{200}Hg were detected in the HFL, HGYL, NCL, and SCS. DGM isotopes in BHS, YS, and ECS were collected for more than 12 h, and it is not allowed to make comparison between the day and night.

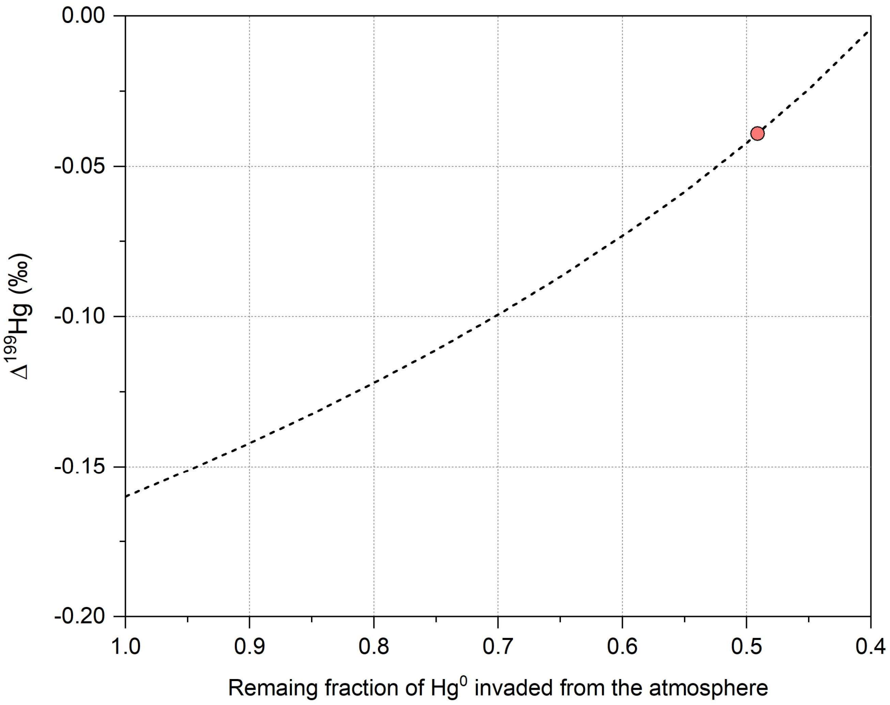

Fig. S6. Estimated Δ^{199}Hg value of aqueous Hg^0 invaded from the atmosphere as a function of Hg^0 oxidation based on a Rayleigh model (dash line): $\ln(\Delta^{199}Hg\times10^{-3}+1)=\ln(\Delta^{199}Hg_i\times10^{-3}+1) + E^{199}Hg_{oxidation}\times10^{-3}\times\ln(f)$. $\Delta^{199}Hg_i$ is the initial Δ^{199}Hg of atmospheric GEM (-0.16‰) measured in this study; $E^{199}Hg_{oxidation}$ is the odd-MIF enrichment factor during aqueous Hg^0 oxidation [25]. Orange dot is the predicted Δ^{199}Hg value of the remaining aqueous Hg^0 directly invaded from the atmosphere using the oxidation rate of 51% estimated in Fig. 4a.

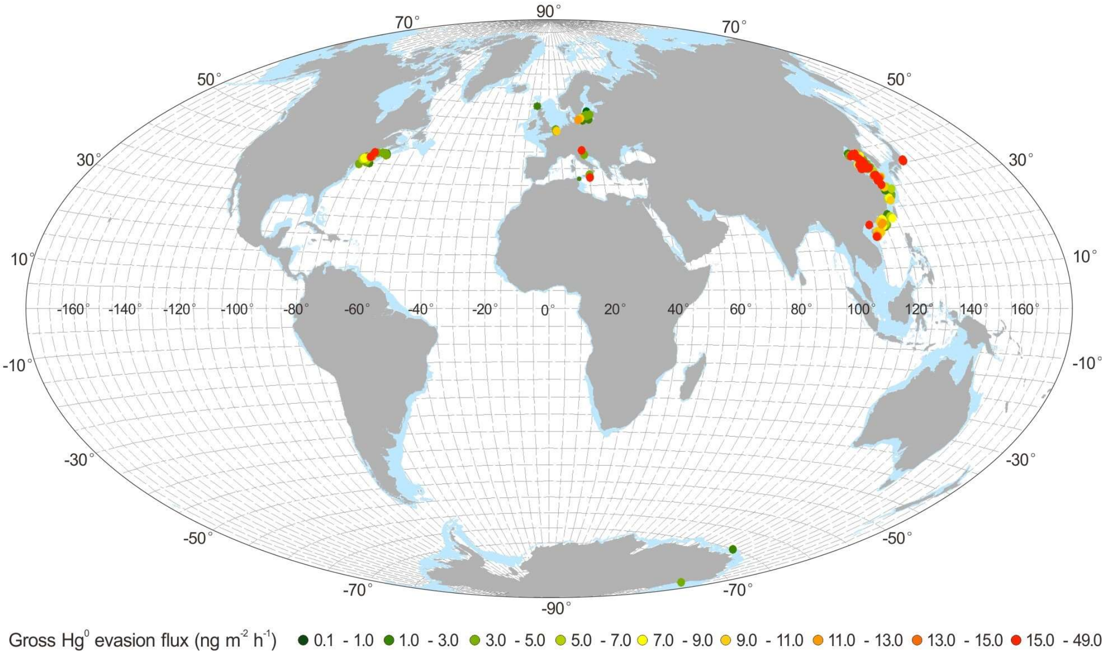

Fig. S7. Gross Hg^0 evasion fluxes in global coastal oceans. light blue color shaded areas represent the global coastal ocean regions[26]. Flux data are from literature (Table S2)

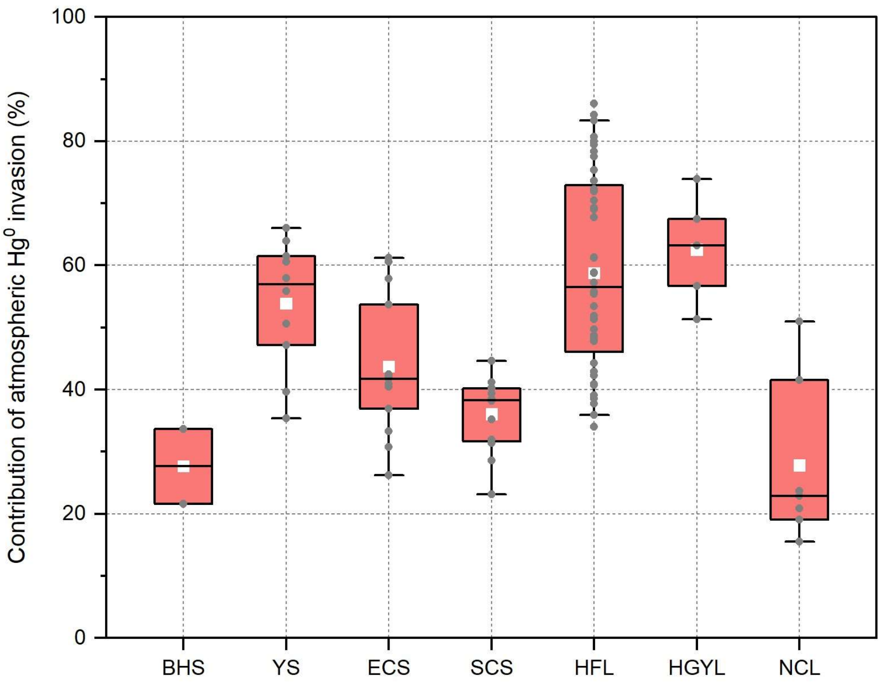

Fig. S8. Fractions of DGM sourced from atmospheric Hg^0 directly in the seas and lakes. The white square and black line in the box are the mean and median, respectively. The boxes, whiskers, and gray dots represent the IQR, 5th-95th range, and individual values out of the IQR, respectively.

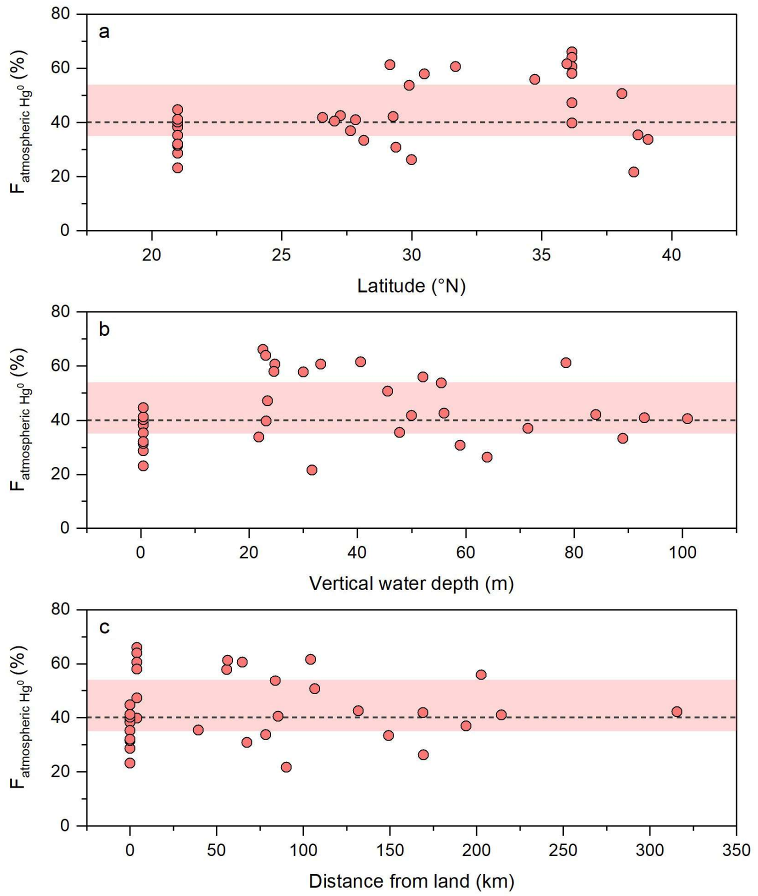

Fig. S9. Correlations between direct atmospheric Hg^0 contributions to DGM and potential factors. (a), (b), and (c) atmospheric Hg^0 contributions versus latitude, vertical water depth, and distance from land, respectively. Vertical water depth was measured by the cruise. Distance from land was calculated using an ArcGIS tool. The black dash line and light orange color shaded areas represent the median and IQR of direct atmospheric Hg^0 contributions, respectively. All the correlations were statistically insignificant (ANOVA, $p > 0.05$ for both).

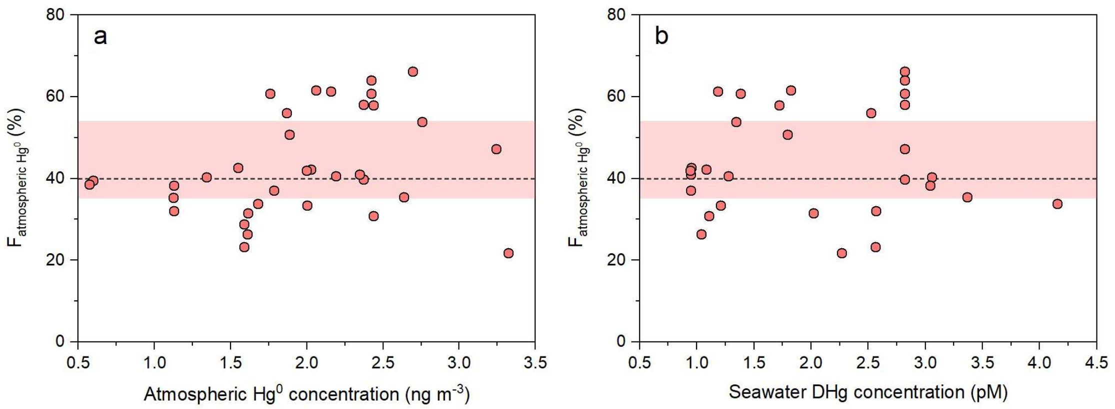

Fig. S10. Relationship between the direct atmospheric Hg^0 contribution and atmospheric Hg^0 concentration and seawater DHg concentration. (a) atmospheric Hg^0 contribution versus atmospheric Hg^0 concentration, and b atmospheric Hg^0 contribution versus seawater DHg concentration. The black dash line and light orange color shaded area indicate the mean and IQR of atmospheric Hg^0 contributions, respectively. No significant correlation was observed between atmospheric Hg^0 contribution and atmospheric Hg^0 concentration and seawater DHg concentration (ANOVA, $p > 0.05$ for both).

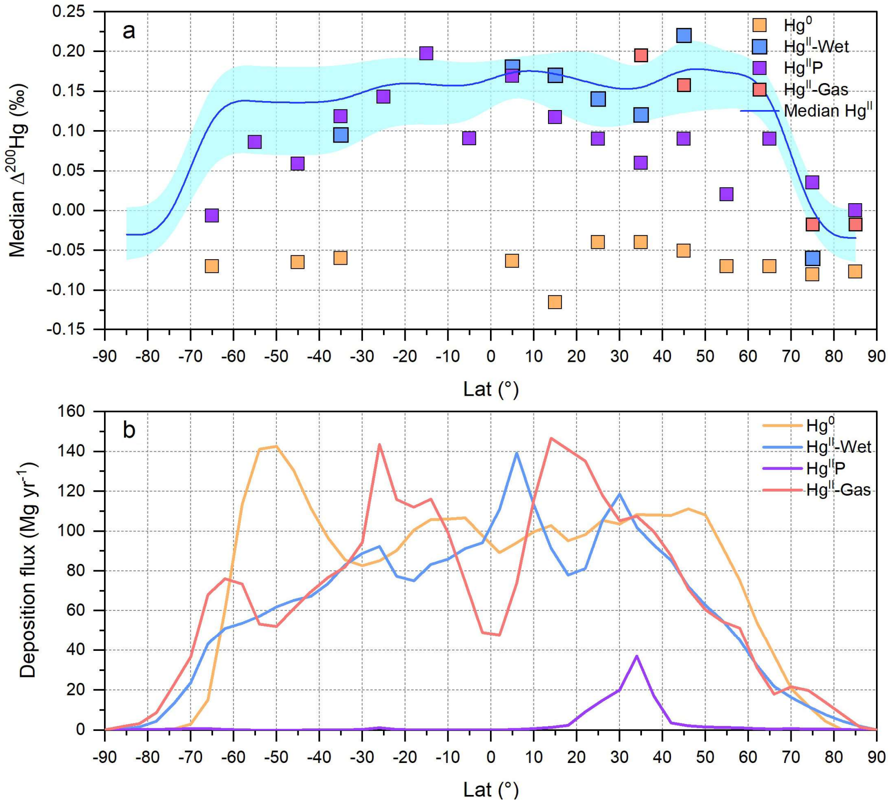

Fig. S11. Latitudinal variations in speciated atmospheric Hg deposition fluxes and Δ^{200}Hg. (a) and (b) mean Δ^{200}Hg and gross deposition fluxes in 5° latitude bins, respectively. Mean Δ^{200}Hg of atmospheric Hg^0, precipitation Hg^{II} (Hg^{II}-Wet), particulate Hg^{II} (Hg^{II}P), and gaseous Hg^{II} (Hg^{II} -Gas) in each 5° latitude bins were calculated using the data in literature (Table S4). Blue line and light bule color shaded area represent the combined median and IQR of atmospheric Hg^{II} deposition, respectively, which were calculated using equation (12) and (13) in Text S9. Latitudinal gross deposition fluxes at 5° intervals are from Shah et al.[18].

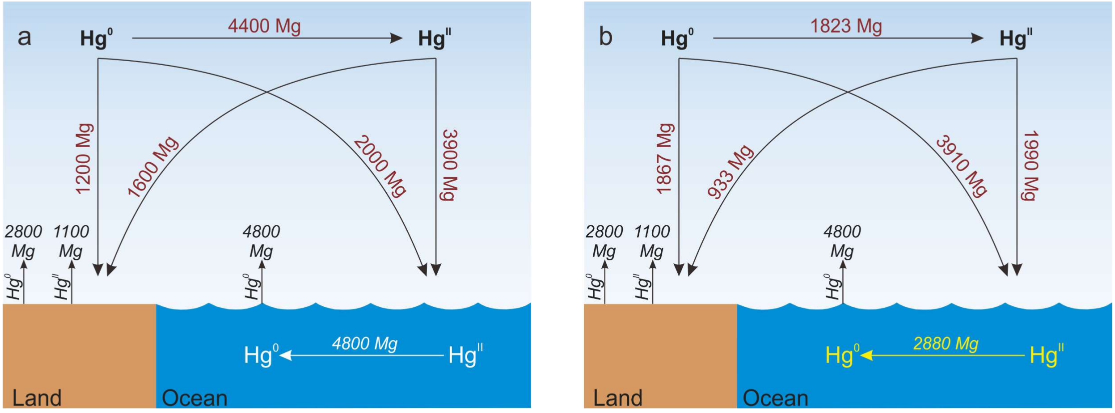

Fig. S12. Updated global atmospheric Hg budget. a and b atmospheric Hg budget in Shah et al. [18] and updated based on the new DGM isotope finding in this study. Atmospheric Hg emission and deposition fluxes in (a) are from Shah et al. [18], and the Hg^{II} emission flux over land is adjusted to be 1100 Mg yr^{-1} to balance the global atmospheric Hg^{II} deposition flux. Atmospheric Hg^0 and Hg^{II} deposition fluxes over ocean and land in (b) are calculated using the total atmospheric Hg deposition flux (5900 and 2800 Mg yr^{-1}, respectively) over these two ecosystems and the estimated gross Hg^0:Hg^{II} deposition ratio in this study, respectively. The net Hg^{II} reduction flux in (b) is estimated based on the relative contribution (60%) of Hg^{II} reduction to DGM in seawater and gross DGM evasion flux of 4800 Mg yr^{-1} from ocean [18].

Supplementary Data references:

1. Gardfeldt K, Sommar J, Ferrara R *et al.* Evasion of mercury from coastal and open waters of the Atlantic Ocean and the Mediterranean Sea. *Atmos Environ*. 2003; **37**: S73-S84. doi: Doi 10.1016/S1352-2310(03)00238-3
2. Mason RP, Sullivan KA. The distribution and speciation of mercury in the South and equatorial Atlantic. *Deep-Sea Res Pt Ii*. 1999; **46**(5): 937-956. doi: Doi 10.1016/S0967-0645(99)00010-7
3. Zhang H, Wu X, Deng Q *et al.* Extraction of ultratrace dissolved gaseous mercury and reactive mercury in natural freshwater for stable isotope analysis. *J Anal Atom Spectrom*. 2021; **36**(9): 1921-1932. doi: 10.1039/D1JA00212K
4. USEPA. Method 1631, Revision E: Mercury in Water by Oxidation, Purge and Trap, and Cold Vapor Atomic Fluorescence Spectrometry. *United States Environmental Protection Agency*. 2002: 10-46.
5. Li K, Lin C-J, Yuan W *et al.* An improved method for recovering and preconcentrating mercury in natural water samples for stable isotope analysis. *J Anal Atom Spectrom*. 2019; **34**(11): 2303-2313. doi: 10.1039/C9JA00174C
6. Fu XW, Heimburger LE, Sonke JE. Collection of atmospheric gaseous mercury for stable isotope analysis using iodine- and chlorine-impregnated activated carbon traps. *J Anal Atom Spectrom*. 2014; **29**(5): 841-852. doi: Doi 10.1039/C3ja50356a
7. Luippold A, Gustin MS, Dunham-Cheatham SM *et al.* Use of Multiple Lines of Evidence to Understand Reactive Mercury Concentrations and Chemistry in Hawai'i, Nevada, Maryland, and Utah, USA. *Environmental Science & Technology*. 2020; **54**(13): 7922-7931. doi: 10.1021/acs.est.0c02283
8. Miller MB, Howard DA, Pierce AM *et al.* Atmospheric reactive mercury concentrations in coastal Australia and the Southern Ocean. *Sci Total Environ*. 2021; **751**. doi: ARTN 141681
10.1016/j.scitotenv.2020.141681
9. He Y, Mason RP. Comparison of reactive gaseous mercury measured by KCl-coated denuders and cation exchange membranes during the Pacific GEOTRACES GP15 expedition. *Atmos Environ*. 2021; **244**: 117973. doi: https://doi.org/10.1016/j.atmosenv.2020.117973
10. Fu XW, Jiskra M, Yang X *et al.* Mass-Independent Fractionation of Even and Odd Mercury Isotopes during Atmospheric Mercury Redox Reactions. *Environmental Science & Technology*. 2021; **55**(14): 10164-10174. doi: 10.1021/acs.est.1c02568
11. Jiskra M, Heimbürger-Boavida L-E, Desgranges M-M *et al.* Mercury stable isotopes constrain atmospheric sources to the ocean. *Nature*. 2021; **597**(7878): 678-682. doi: 10.1038/s41586-021-03859-8
12. Blum JD, Bergquist BA. Reporting of variations in the natural isotopic composition of mercury. *Anal Bioanal Chem*. 2007; **388**(2): 353-359. doi: DOI 10.1007/s00216-007-1236-9
13. Blum JD, Johnson MW. Recent Developments in Mercury Stable Isotope Analysis in Non-Traditional Stable Isotopes. In: Teng FZ, Watkins J, Dauphas N (eds.). De Gruyter; 2017. 733-757.
14. Ci Z, Zhang X, Yin Y *et al.* Mercury Redox Chemistry in Waters of the Eastern Asian Seas: From Polluted Coast to Clean Open Ocean. *Environmental Science & Technology*. 2016; **50**(5): 2371-2380. doi: 10.1021/acs.est.5b05372
15. Amyot M, Mierle G, Lean D *et al.* Effect of solar radiation on the formation of dissolved gaseous mercury in temperate lakes. *Geochim Cosmochim Ac*. 1997; **61**(5): 975-987. doi: Doi 10.1016/S0016-7037(96)00390-0

16. O'Driscoll NJ, Vost E, Mann E *et al.* Mercury photoreduction and photooxidation in lakes: Effects of filtration and dissolved organic carbon concentration. *J Environ Sci*. 2018; **68**: 151-159. doi: https://doi.org/10.1016/j.jes.2017.12.010
17. Soerensen AL, Sunderland EM, Holmes CD *et al.* An Improved Global Model for Air-Sea Exchange of Mercury: High Concentrations over the North Atlantic. *Environmental Science & Technology*. 2010; **44**(22): 8574-8580. doi: Doi 10.1021/Es102032g
18. Shah V, Jacob DJ, Thackray CP *et al.* Improved Mechanistic Model of the Atmospheric Redox Chemistry of Mercury. *Environmental Science & Technology*. 2021; **55**(21): 14445-14456. doi: 10.1021/acs.est.1c03160
19. Song Z, Huang S, Zhang P *et al.* Isotope Data Constrains Redox Chemistry of Atmospheric Mercury. *Environmental Science & Technology*. 2024; **58**(30): 13307-13317. doi: 10.1021/acs.est.4c02600
20. Sprovieri F, Pirrone N, Bencardino M *et al.* Atmospheric mercury concentrations observed at ground-based monitoring sites globally distributed in the framework of the GMOS network. *Atmos Chem Phys*. 2016; **16**(18): 11915-11935.
21. Motta LC, Kritee K, Blum JD *et al.* Mercury Isotope Fractionation during the Photochemical Reduction of Hg(II) Coordinated with Organic Ligands. *The Journal of Physical Chemistry A*. 2020; **124**(14): 2842-2853. doi: 10.1021/acs.jpca.9b06308
22. Zheng W, Hintelmann H. Mercury isotope fractionation during photoreduction in natural water is controlled by its Hg/DOC ratio. *Geochim Cosmochim Ac*. 2009; **73**(22): 6704-6715. doi: DOI 10.1016/j.gca.2009.08.016
23. Kritee K, Blum JD, Barkay T. Mercury Stable Isotope Fractionation during Reduction of Hg(II) by Different Microbial Pathways. *Environmental Science & Technology*. 2008; **42**(24): 9171-9177. doi: Doi 10.1021/Es801591k
24. Zheng W, Foucher D, Hintelmann H. Mercury isotope fractionation during volatilization of Hg(0) from solution into the gas phase. *J Anal Atom Spectrom*. 2007; **22**(9): 1097-1104. doi: Doi 10.1039/B705677j
25. Zheng W, Demers JD, Lu X *et al.* Mercury Stable Isotope Fractionation during Abiotic Dark Oxidation in the Presence of Thiols and Natural Organic Matter. *Environmental Science & Technology*. 2019; **53**(4): 1853-1862. doi: 10.1021/acs.est.8b05047
26. Laruelle GG, Dürr HH, Lauerwald R *et al.* Global multi-scale segmentation of continental and coastal waters from the watersheds to the continental margins. *Hydrol Earth Syst Sci*. 2013; **17**(5): 2029-2051. doi: 10.5194/hess-17-2029-2013
